# “Ecstasy” toxicity to adolescent rats following an acute low binge dose

**DOI:** 10.1186/s40360-016-0070-0

**Published:** 2016-06-28

**Authors:** Armanda Teixeira-Gomes, Vera Marisa Costa, Rita Feio-Azevedo, José Alberto Duarte, Margarida Duarte-Araújo, Eduarda Fernandes, Maria de Lourdes Bastos, Félix Carvalho, João Paulo Capela

**Affiliations:** UCIBIO-REQUIMTE (Rede de Química e Tecnologia), Laboratório de Toxicologia, Departamento de Ciências Biológicas, Faculdade de Farmácia, Universidade do Porto, Rua de Jorge Viterbo Ferreira, 228, 4050-313 Porto, Portugal; CIAFEL, Faculdade de Desporto, Universidade do Porto, Porto, Portugal; Biotério do Instituto de Ciências Biomédicas de Abel Salazar (ICBAS), Universidade do Porto, Porto, Portugal; UCIBIO-REQUIMTE, Laboratório de Química Aplicada, Departamento de Química, Faculdade de Farmácia, Universidade do Porto, Porto, Portugal; FP-ENAS (Unidade de Investigação UFP em Energia, Ambiente e Saúde), CEBIMED (Centro de Estudos em Biomedicina), Faculdade de Ciências da Saúde, Universidade Fernando Pessoa, Porto, Portugal

**Keywords:** “Ecstasy”, Adolescent, Peripheral toxicity, Brain, Hyperthermia, Oxidative stress

## Abstract

**Background:**

3,4-Methylenedioxymethamphetamine (MDMA or “ecstasy”) is a worldwide drug of abuse commonly used by adolescents. Most reports focus on MDMA’s neurotoxicity and use high doses in adult animals, meanwhile studies in adolescents are scarce. We aimed to assess in rats the acute MDMA toxicity to the brain and peripheral organs using a binge dose scheme that tries to simulate human adolescent abuse.

**Methods:**

Adolescent rats (postnatal day 40) received three 5 mg/kg doses of MDMA (estimated equivalent to two/three pills in a 50 kg adolescent), intraperitoneally, every 2 h, while controls received saline. After 24 h animal sacrifice took place and collection of brain areas (cerebellum, hippocampus, frontal cortex and striatum) and peripheral organs (liver, heart and kidneys) occurred.

**Results:**

Significant hyperthermia was observed after the second and third MDMA doses, with mean increases of 1 °C as it occurs in the human scenario. MDMA promoted ATP levels fall in the frontal cortex. No brain oxidative stress-related changes were observed after MDMA. MDMA-treated rat organs revealed significant histological tissue alterations including vascular congestion, but no signs of apoptosis or necrosis were found, which was corroborated by the lack of changes in plasma biomarkers and tissue caspases. In peripheral organs, MDMA did not affect significantly protein carbonylation, glutathione, or ATP levels, but liver presented a higher vulnerability as MDMA promoted an increase in quinoprotein levels.

**Conclusions:**

Adolescent rats exposed to a moderate MDMA dose, presented hyperthermia and acute tissue damage to peripheral organs without signs of brain oxidative stress.

## Background

Over the past decades, amphetamine-type psychostimulants, such as 3,4-methylenedioxymethamphetamine (MDMA, “ecstasy”) became widely used as recreational drugs by adolescents and young people around the world [1, 2]. According to the United Nations Office of Drugs and Crime, the estimated global users of MDMA ranged from 9.4 to 28.2 million in 2012 [3]. In Europe, 2.0 of the 2.5 million past-year “ecstasy” users in 2010 were estimated to be 15–34 years old; and, in the United States of America, 2.5 of the estimated 2.6 million people who had used MDMA in the past year were aged from 14 to 34 years [3]. Recreational use of MDMA is frequently done by binge administration that is a pattern of several administrations over a short time period [4–6].

MDMA is a drug frequently used by adolescents and therefore we need to understand the consequences of MDMA exposure at this age through the administration of doses and schemes more close to the human situation. Interestingly, the vast majority of studies tend to use adult animals for studying MDMA toxicity. One reason for this might be that the majority of studies focus on MDMA’s neurotoxicity and neurotoxic effects seem to be less severe in younger animals when compared to adult animals [2]. MDMA administration to adult laboratory animals was shown to promote long-term depletion of monoamine neurotransmitter content, damage to the nerve terminals, neuronal cell death and long-lasting cognitive impairments [1, 2]. Notably, studies conducted with adolescent animals have shown that MDMA exposure to neurotoxic doses leads to deficits in the serotonergic system [2] and also late changes in memory and learning abilities [7, 8]. Investigation on adolescent laboratory animals is of extreme importance, given that studies with human adolescents have ethical barriers, making extremely difficult to enrol this population in studies that evaluate drug abuse toxicity. Nonetheless, studies with young adults reported deficits in the serotonergic system [9, 10], decreased grey matter concentration [11] and cognitive deficits [12, 13] following MDMA abuse.

In order to produce neurotoxicity in laboratory animals’ researchers need a certain dose of MDMA, as neurotoxicity was shown to be dose-dependent [1, 2]. MDMA neurotoxic doses in rats generally range from 20 to 40 mg/kg on a session of exposure, but the cumulative dose can be much higher when treatments take place through several days [1]. These high doses, while important for studying neurotoxic related events, do not match the typical MDMA user profile, as they tend to be extremely high and correlate only to a high-intensity abuser. One can try to extrapolate the dose used in animals to the equivalent in humans using the allometric scaling principles, for instance: *Human dose (mg/kg) = animal dose (mg/kg) × (animal weight/human weight)*^*1/4*^ [14]. This method does not account for the differences in MDMA metabolism or administration route between humans and rats, but is certainly of great value for an approximate extrapolation. According to this relation a dose of 40 mg/kg in adult rats, used in a daily session, is equivalent to approximately 700 mg in a human with 70 kg. According to the latest European Union report MDMA pills range from 57 to 102 mg [15], and therefore that dose would mean an average intake of more than seven pills in a single session, a rather extreme scenario. The binge dosing pattern that we selected for this study [three times 5 mg/kg MDMA, intraperitoneal (i.p.), every 2 h] was reported to promote long-term serotonergic neurotoxicity in adult Wistar rats (10 week old) [16]. We have conducted experiments that proved that this MDMA regimen did not induce serotonergic toxicity 7 days following MDMA administration to adolescent (PND 40) Wistar rats, as no 5-HT depletion could be found in any of the four brain areas evaluated (manuscript being prepared for submission). Using the mentioned formula based on allometric scaling principles, we estimate that a cumulative MDMA dose of 15 mg/kg in adolescent rats is estimated to be equivalent in a 50 kg human adolescent to 170 mg. “Ecstasy” typical abusers generally take more than one tablet *per* session, ranging from two to four tablets in accordance with the binge-dosing pattern [5, 6]. Therefore, and in accordance with the last European Union report on drugs, we estimate that the dose used in our protocol is equivalent to the intake of two to three pills by adolescents using the binge-dosing pattern. Accordingly, the current paradigm of exposure to adolescent animals tries to mimic the dose schedule used by human adolescents. For that reason studies using more moderate doses that closely match the typical MDMA user are needed.

The majority of studies focus on MDMA’s neurotoxicity and neglect the toxicity of this drug to the peripheral organs. In vivo and in vitro studies reported decreases in adenosine 5′-triphosphate (ATP) [17–19] and in glutathione levels [20–22], as well as increases in protein carbonylation [23] in the peripheral organs following exposure to MDMA or its metabolites. Additionally, MDMA abuse was associated with histopathological evidences of toxicity to the liver, heart and kidneys in humans [24, 25]. Most studies that evaluate human “ecstasy” toxicity to peripheral organs are conducted following fatal outcomes or after admission to emergency rooms. Studies are lacking regarding the evaluation of MDMA-induced damage to organs following lower doses. Moreover, at this point, studies in adolescent animal models regarding peripheral toxicity using moderate MDMA doses have not been done.

In our study, we aimed to investigate MDMA toxic effects to the brain and peripheral organs in an adolescent rat, using a moderate dose by binge administration of MDMA, which mimics better the dose schedule of “ecstasy” users. To the best of our knowledge, this is the first study in an adolescent rat model to evaluate simultaneously in three peripheral organs oxidative stress parameters and histological damage.

## Methods

### Materials

Folin–Ciocalteu reagent, cupper (II) sulphate, dimethyl sulfoxide, disodium phosphate, ethylenediaminetetraacetic acid (EDTA), perchloric acid, potassium bicarbonate (KHCO_3_), sodium hydroxide, sodium carbonate, magnesium chloride, potassium dihydrogen phosphate and magnesium sulphate were purchased from Merck (Darmstadt, Germany). Potassium sodium tartrate was purchased from Fluka (Buchs SG, Switzerland), and sodium phosphate monobasic from Panreac (Barcelona, Spain). Phosphate buffered saline solution (PBS) was obtained from Biochrom (Berlin, Germany). From VWR (Leuven, Belgium) was purchased sodium dodecyl sulphate and sodium chloride (NaCl). Meanwhile, 4-(2-hydroxyethyl)piperazine-1-ethanesulfonic acid (HEPES), xylene and methanol were obtained from Fisher Scientific (Loughborough, UK). Isoflurane (Isoflo®) was purchased from Abbott (IL, USA). Lidocaine 25 mg/g + Prilocaine 25 mg/g (EMLA®) was acquired from AstraZeneca (London, UK). The fluorescent peptide substrates for the caspase activity assays were acquired from Peptanova (Sandhausen, Germany). For western blot, dinitropenhyl-KLH rabbit IgG antibody was purchased from Invitrogen/Life Technologies (NY, USA), meanwhile 0.45 μm Amersham Protran nitrocellulose blotting membrane and horseradish peroxidase (HRP) conjugated anti-rabbit antibody were acquired from GE Healthcare Bio-Sciences (PA, USA). Histofluid was purchased from Marienfeld (Lauda-Königshofen, Germany), Harris hematoxylin was obtained at Harris Surgipath (IL, USA), and eosin 1 % solution from Biostain (Traralgon, Australia). From Bio-Rad Laboratories (CA, USA) was acquired the Bio-Rad DC protein assay kit and the Clarity Western ECL reagent. All the other reagents used were purchased from Sigma-Aldrich (MO, USA). MDMA (HCl salt) was extracted and purified from MDMA tablets, which were high in purity, and were kindly provided by the Portuguese Criminal Police Department. As we have previously detailed, the extracted salt was fully characterized by mass spectrometry and nuclear magnetic resonance, yielding a high degree of purity that was superior to 95 % [26].

### Animals

Fourteen adolescent male Wistar rats at postnatal day (PND) 40 and weighing in average 115 ± 11 g were born at the Institute for Biomedical Sciences Abel Salazar-University of Porto (ICBAS-UP) animal facilities. Animals were housed in a controlled environment [temperature of 22.0 ± 2.0 °C, 40 % humidity and 12 hours (h) light/dark cycles]. Animals had ad libitum access to food and water and throughout the experimental period had permanent veterinary supervision.

All procedures were performed as to give the proper animal care, to reduce suffering and stress. Experimental animal procedures were in agreement with the European Council Directive (2010/63/EU) guidelines that where transposed into Portuguese law (Decreto-Lei n.° 113/2013, de 7 de Agosto). Additionally, the experiments were conducted with the approval of the Ethical Committee of the Faculty of Pharmacy, University of Porto (process n° 17/03/2014) and approved by the Portuguese National Authority for Animal Health (General Directory of Veterinary Medicine) (process n° 0421/000/000/2015).

### Experimental protocol

Three days before the experiment, the dorsocervical region was trichotomised and a local anaesthetic (Lidocaine 25 mg/g + Prilocaine 25 mg/g) was allowed to act for about 60 minutes (min). Then each animal was subjected to a brief inhalatory anaesthesia with isoflurane to perform a subcutaneous insertion of a temperature transponder (BioMedic Data Systems Inc) with minimum animal discomfort. This transponder ensures precise core body temperature measurements throughout the entire experimental period, as we reported before [27]. Prior to MDMA administration, animals were maintained in groups allowing conspecific social interactions. In the administration day and for the next 24 h, animals were housed individually.

Animals were randomly assigned to the two experimental groups: control (*n* = 7) and MDMA-treated (*n* = 7). We selected this sample size per group based on a pilot study that we performed. Additionally, seven animals per treatment allow the mathematical detection of differences among means of two standard deviations (assuming a 5 % significance level in a t-test and a 90 % power) [28]. For a full explanation on these statistical calculations please see reference [28]. Additionally, sample size took into consideration ethical reasons related to animal welfare in a pre-clinical study conducted with drugs of abuse, such as the present one. The group treated with MDMA received three doses of 5 mg/kg MDMA salt, i.p., every 2 h (total cumulative dose of 15 mg/kg). MDMA was prepared at the day of use in a concentration of 2.5 mg/mL in sterile NaCl 0.9 % (saline solution). Control animals received saline solution at the same schedule and using equivalent injection volumes of treated animals. Rats’ temperature was monitored and registered every 15 min for a total of 7 h, after the first dose. Food and water consumption, as well as animal weight were evaluated before the first injection and at the next day. Twenty-four hours following the first MDMA administration adolescent rats were sacrificed.

### Brain and organ tissue collection

Animals were anesthetized and euthanized with the volatile anaesthetic, isoflurane. Before decapitation, blood was collected from the inferior vena cava. Following sacrifice, brain areas (cerebellum, hippocampus, frontal cortex and striatum) and peripheral organs (liver, heart and kidneys) were collected. The collection of the different brain regions was performed in agreement to a rat brain atlas [29], and the dissection techniques were conducted in accordance to a previous work [30]. The brain and peripheral organs were also weighted.

#### Brain tissue treatment

Samples of brain areas from the right hemisphere were collected in RIPA buffer supplemented with protease inhibitors [0.1 % sodium dodecyl sulphate, 1 % Triton X-100, 0.5 % sodium deoxycholate, 150 mM NaCl, 50 mM Tris, 1 mM sodium fluoride, 1 mM sodium metavanadate, 0.25 mM phenylmethanesulfonyl fluoride (PMSF), proteases inhibitor cocktail from Sigma, pH = 8.0]. Then brain samples were homogenized while kept on ice using a sonicator (20 seconds, continuously) and centrifugation followed (16,000 *g*, 15 min at 4 °C). The supernatants were separated for the protein carbonylation and quinoprotein analysis. Meanwhile, brain samples obtained from the left side of each hemisphere were placed in 5 % perchloric acid and homogenized in a sonicator (20 seconds, continuously), while tubes were kept on ice. Centrifugation was followed (16,000 *g*, 10 min at 4 °C), and the resulting supernatants separated for ATP and glutathione determinations. The sample pellets were stored for protein determination. All samples were frozen at −80 °C until further analysis.

#### Organ tissue treatment

Small sections (5 mm^3^) of liver (from different lobes), heart (apex) and kidneys (including cortex and medulla) were fixed during 24 h using a 4 % paraformaldehyde solution in PBS, and further processed for qualitative histologic analysis. A section of liver, heart and kidneys was collected for caspases activity assay in caspase lysis buffer (0.5 % Triton X-100, 25 mM HEPES, 5 mM magnesium chloride, 1 mM EDTA, 5 mM dithiothreitol, and 1 mM PMSF, pH = 7.4). Another section was collected in complete RIPA buffer supplemented with protease inhibitors, and homogenized using a sonicator (30 seconds, continuously), while tubes were kept on ice. Centrifugation was followed (2376 *g*, 10 min at 4 °C) and the supernatants were collected for the quinoprotein and protein carbonylation determinations. Samples in caspase lysis buffer or in RIPA buffer were stored at −80 °C until further analysis. Another section of each organ was collected and homogenized in an Ultra-Turrax (samples diluted in 0.1 M potassium dihydrogen phosphate solution, pH = 7.4). One portion of homogenate was stored at −20 °C for protein quantification. Other portion of homogenate was added to perchloric acid 5 % solution and centrifuged (16,000 *g*, 10 min at 4 °C). The resultant supernatants were then stored for ATP and total glutathione (GSHt) or oxidized glutathione (GSSG) biochemical determinations. Samples for reduced glutathione (GSH)/GSSG analysis were stored at −20 °C, meanwhile those for ATP quantification were frozen at −80 °C.

### Measurement of plasma biomarkers

The levels of aspartate aminotransferase (AST) and alanine aminotransferase (ALT), as well as total creatine kinase (CK) and creatine kinase-MB (CK-MB), were determined in blood plasma as previously described by our group [31]. The enzymatic assays were conducted according to the manufacturer’s instructions in the apparatus ABX Pentra 400 (Kyoto, Japan).

### Assessment of ATP levels

ATP levels were quantified by a bioluminescent assay using the luciferin-luciferase system, as described in detail in previous works [26, 32]. The results are presented in nmol of ATP *per* mg of protein.

### Measurement of GSHt, GSH and GSSG

The GSHt or GSSG levels were evaluated by the 5,5′-dithiobis(2-nitrobenzoic acid)-GSSG reductase recycling assay, as previously we described in detail [26, 32]. GSH levels were calculated by the equation: GSH = GSHt – 2 × GSSG. Levels of GSHt, GSSG and GSH were normalized to the protein content (results presented in nmol GSH *per* mg protein or nmol GSSG *per* mg protein).

### Protein carbonylation assay

Protein carbonylation was determined as we previously reported [33], with minor modifications. Samples (0.1 mg protein/mL) reacted with 2,4-dinitrophenylhydrazine (0.2 μg) and were loaded into nitrocellulose membranes (Hybond ECL, Amersham Pharmacia Biotech) in a slot blot device. After washing steps, membranes were exposed to the primary antibody (rabbit polyclonal anti-DNP, 1:1000) overnight at 4 °C. Incubation at room temperature with the secondary antibody (anti-rabbit IgG-peroxidase, 1:2000, 1 h) was followed. Exposure to Clarity™ Western ECL Substrate (Bio-Rad, CA, USA) was used to visualize membrane bands. Digital images were obtained and treated with Molecular Imager® ChemiDocTM XRS+ System (Bio-Rad, CA, USA). Membranes were analysed with Image Lab Software (Bio-Rad, CA, USA) and results expressed as % of optical density control values.

### Assessment of quinoproteins

The protein-bound quinones were determined using nitrotetrazolium blue chloride/glycinate colorimetric assay, as previously described [26]. For the assay, lysates in RIPA buffer were used, and the reaction mixture of brain samples contained 50 μg of protein, meanwhile peripheral organs samples contained 25 μg of protein.

### Histological tissue procedures

Sections of liver, heart, and kidneys were prepared, and analysed, as previously described by our group [31]. Following fixation in 4 % paraformaldehyde and paraffin embedding, sections with 5 μm were mounted on silane-coated slides (Sigma, S4651-72EA), and stained with hematoxylin/eosin following routine procedures. Samples were photographed and analysed in a light microscope (Carl Zeiss Imager A1 attached to a digital camera AxioCam MRc 5, Oberkochen, Germany).

### Assessment of caspases-3, -9 and -8 activities

A fluorescent assay for tissues was used to determine each caspase activity in the liver, heart and kidneys of the animals, as previously described by our group [31]. The following fluorescent peptide substrates were used: Ac-DMQD-AMC, for caspase-3, Ac-IETD-AMC, for caspase-8, and Ac-LEHD-AMC for caspase-9. Caspase activity was expressed in fluorescent units *per* μg of protein (FU/μg protein).

### Protein sample quantification

The protein content of samples in RIPA or caspase lysis buffer was quantified using the DC Protein Assay kit (Bio-Rad, CA, USA). For other samples, proteins were quantified by the Lowry method [34].

### Statistical analysis

**Fig. 1:**
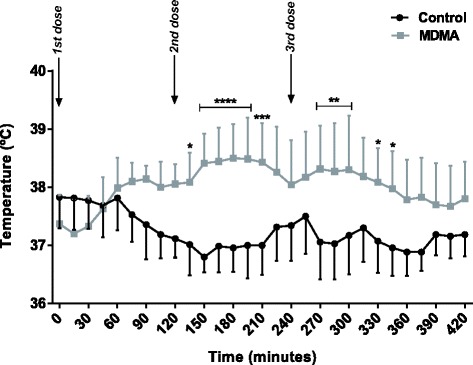
Temperature monitoring of adolescent rats after exposure to three doses of NaCl 0.9 % i.p. (control) or 5 mg/kg MDMA i.p. (MDMA-treated) during 7 h. Results in degrees Celsius (°C) are presented as mean ± standard deviation, from seven animals in each group. Statistical comparisons were made using two-way ANOVA repeated measurements followed by the Bonferroni post hoc test (*p <0.05, **p <0.01, ***p <0.001 and ****p <0.0001 treatment vs. control)

Results in tables and graphics are presented as mean ± standard deviation. Statistical analysis was conducted using GraphPad Prism version 6 (GraphPad Software, La Jolla California, USA). The Shapiro–Wilk normality test was conducted before group comparison. For data where two groups were compared, the t-test was used for a normal distribution or the Mann-Whitney Rank Sum test when data did not follow a normal distribution. Statistical analysis of the temperature, included in Fig. 1, was conducted by a two-way analysis of variance (ANOVA) with repeated measurements, followed by Bonferroni post-hoc test, once a significant *p* was obtained. Statistical significance was accepted at *p* values less than 0.05.

## Results

### MDMA evoked hyperthermia, but no changes in body weight, food or water intake

Three MDMA doses were given to the animals (each dose 5 mg/kg i.p., every 2 h) and the first dose did not cause significant changes in body temperature (Fig. 1). After the second dose of MDMA, rats had a significantly higher body temperature when compared to the control group (*p* <0.0001). Temperature persisted significantly higher for almost 2 h after the third dose in treated animals. The temperature of MDMA-treated animals never surpassed 39 °C even after the third MDMA dose (Fig. 1). The temperature was also measured 24 h post-MDMA binge administration and no differences were observed in the temperature among controls and MDMA-treated rats (data not shown).

Recordings of body weight gain, food or water intake before and 24 h after exposure showed no differences among control and MDMA-treated rats (data not shown). In fact, there was a slight decrease in the body weight of animals in both groups, possibly as a result of the animal stress due to the manipulation. The food consumption was similar in both control and MDMA-treated animals. However, there was a tendency for an increased water intake in animals that received MDMA, but it was not statistically significant (data not shown).

### Liver, heart and kidneys weights were not changed

Each core organ (liver, heart, kidneys, and brain) had their weight registered and the weight ratio of each organ was taken to brain weight. No significant differences were observed between MDMA-treated rats and the control group for all collected organs (Table 1).

**Table 1:** Organ weight in control and MDMA–treated groups

	Control	MDMA
Heart weight/Brain weight ratio	0.33 ± 0.03	0.32 ± 0.05
Kidneys weight/Brain weight ratio	0.69 ± 0.03	0.66 ± 0.07
Liver weight/Brain weight ratio	3.42 ± 0.17	3.15 ± 0.36

Liver weight/brain weight ratio, heart weight/brain weight ratio, and kidneys weight/brain weight ratio of control and MDMA-treated rats. Results are presented as mean ± standard deviation, and were obtained from seven animals in each group. The mean brain weight of control animals was 1.57 ± 0.04, and of MDMA-treated was 1.63 ± 0.05. Statistical comparisons were made using the t-test for the heart weight/brain weight ratio and the Mann-Whitney Rank Sum test for the liver weight/brain weight ratio and kidneys weight/brain weight ratio

### MDMA decreased ATP content in the frontal cortex

**Fig. 2:**
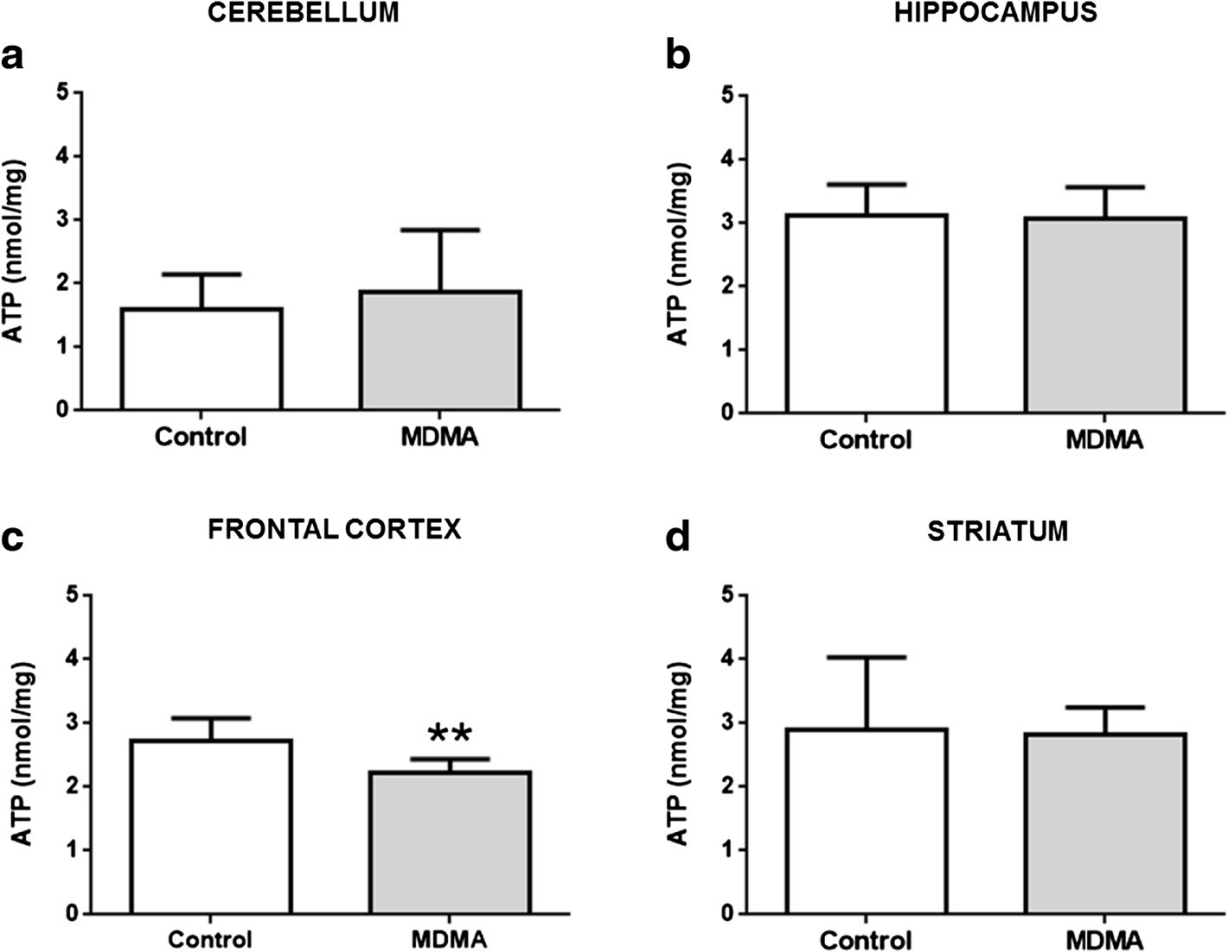
ATP content in the cerebellum (**a**), hippocampus (**b**), frontal cortex (**c**) and striatum (**d**) after MDMA administration to adolescent rats. Results, in nanomol *per* mg of protein (nmol/mg protein), are presented as mean ± standard deviation, and were obtained from seven animals in each group. Statistical comparisons were made using the *t*-test (**p <0.01 treatment vs. control)

Twenty-four hours after MDMA exposure, ATP levels showed no significant changes in cerebellum, hippocampus and striatum, as can be seen in Fig. 2a, b and d, respectively. Importantly, there was a significant decrease in the ATP content of the frontal cortex of the MDMA-treated group (*p* = 0.007) (Fig. 2c).

### No changes in oxidative stress related parameters in the brain areas

There were no significant alterations in the levels of GSHt, GSSG, GSH and GSH/GSSG ratio in the cerebellum, hippocampus, frontal cortex, and striatum of treated rats when compared to controls (Table 2).

**Table 2:** Effect of MDMA administration in oxidative stress related parameters in the four brain areas

Parameter	Control	MDMA
Hippocampus		
GSHt (nmol/mg protein)	21.17 ± 1.22	22.87 ± 3.46
GSH/GSSG ratio	50.30 ± 12.72	50.26 ± 19.37
GSSG (nmol/mg protein)	0.42 ± 0.09	0.41 ± 0.18
GSH (nmol/mg protein)	20.33 ± 1.37	22.05 ± 3.72
Quinoprotein (OD/mg protein)	6.01 ± 0.78	6.09 ± 0.71
Protein carbonylation (% of control)	100.00 ± 28.69	88.01 ± 12.51
Frontal cortex		
GSHt (nmol/mg protein)	18.60 ± 1.78	17.93 ± 1.70
GSH/GSSG ratio	61.44 ± 10.67	68.60 ± 18.71
GSSG (nmol/mg protein)	0.30 ± 0.05	0.27 ± 0.07
GSH (nmol/mg protein)	18.00 ± 1.77	17.39 ± 1.67
Quinoprotein (OD/mg protein)	5.54 ± 0.70	5.73 ± 0.50
Protein carbonylation (% of control)	100.00 ± 11.80	101.15 ± 10.18
Striatum		
GSHt (nmol/mg protein)	20.70 ± 6.14	20.49 ± 4.68
GSH/GSSG ratio	47.49 ± 16.60	56.76 ± 14.98
GSSG (nmol/mg protein)	0.45 ± 0.22	0.35 ± 0.04
GSH (nmol/mg protein)	19.79 ± 5.88	19.78 ± 4.69
Quinoprotein (OD/mg protein)	5.76 ± 0.20	5.78 ± 0.15
Protein carbonylation (% of control)	100.00 ± 53.98	108.84 ± 45.81
Cerebellum		
GSHt (nmol/mg protein)	13.32 ± 0.69	12.34 ± 2.80
GSH/GSSG ratio	25.20 ± 6.52	28.77 ± 12.21
GSSG (nmol/mg protein)	0.51 ± 0.12	0.47 ± 0.21
GSH (nmol/mg protein)	12.29 ± 0.80	11.40 ± 2.67
Quinoprotein (OD/mg protein)	5.93 ± 0.42	5.95 ± 0.29
Protein carbonylation (% of control)	100.00 ± 27.94	95.43 ± 19.88

Total glutathione (GSHt), oxidized glutathione (GSSG), reduced glutathione (GSH), GSH/GSSG ratio levels, quinoprotein levels and protein carbonylation in hippocampus, frontal cortex, striatum and cerebellum of adolescent rats. Data of GSHt, GSSG and GSH levels, in nanomol *per* mg of protein (nmol/mg protein), and the GSH/GSSG ratio are presented as mean ± standard deviation, and were obtained from six to seven animals in each group. Statistical comparisons were made using the t-test for GSHt and GSH levels in hippocampus, frontal cortex and striatum, GSSG levels in cerebellum, hippocampus and frontal cortex, and GSH/GSSG ratio levels in frontal cortex and striatum; the Mann-Whitney Rank Sum test was used for GSHt and GSH levels in cerebellum, GSSG levels in striatum, and GSH/GSSG ratio levels in cerebellum and hippocampus. Results of quinoprotein levels, in optical density *per* mg of protein (OD/mg protein), and protein carbonylation, in percentage of controls (% of control), are presented as mean ± standard deviation, and were obtained from seven animals in each group. Statistical comparisons were made using t-test for the quinoprotein levels in cerebellum and striatum and protein carbonylation in cerebellum, hippocampus and frontal cortex; and Mann-Whitney Rank Sum test for quinoprotein levels in hippocampus and frontal cortex, and for protein carbonylation in striatum

Moreover, MDMA had no influence on quinoprotein levels in the brain areas. In cerebellum, hippocampus, frontal cortex and striatum no differences were found between controls and MDMA-treated animals regarding quinoprotein levels (Table 2).

The levels of protein carbonylation in the cerebellum, hippocampus, frontal cortex and striatum are also presented in Table 2, and there were no significant alterations in protein carbonyl levels in the four brain areas of treated animals.

### No changes in plasma AST, ALT, CK-MB and CK levels

The plasma levels of CK, CK-MB, AST and ALT were measured 24 h after exposure as biomarkers of liver, heart or muscle integrity (Table 3). There were no significant differences in these parameters between control and MDMA-treated animals. The ratio AST/ALT was also calculated, but no significant differences among groups were found (data not shown).

**Table 3:** Effect of MDMA administration in plasma AST, ALT, CK-MB and CK levels

	Control	MDMA
AST (U/L)	39.14 ± 17.53	45.71 ± 19.44
ALT (U/L)	25.00 ± 2.00	28.57 ± 3.82
CK (U/L)	202.57 ± 76.80	200.29 ± 75.54
CK-MB (U/L)	340.14 ± 138.90	305.86 ± 132.91

Plasma levels of total creatine kinase (CK), creatine kinase-MB (CK-MB), aspartate aminotransferase (AST) and alanine aminotransferase (ALT) of control and MDMA-treated rats. Results of CK, CK-MB, AST and ALT plasma levels are presented as mean ± standard deviation, and were obtained from six to seven animals in each group. Statistical comparisons were made using the t-test for CK, CK-MB and AST levels, and the Mann-Whitney Rank Sum test for ALT levels

### MDMA promoted vacuolization, oedema and vascular congestion in the peripheral organs

**Fig. 3:**
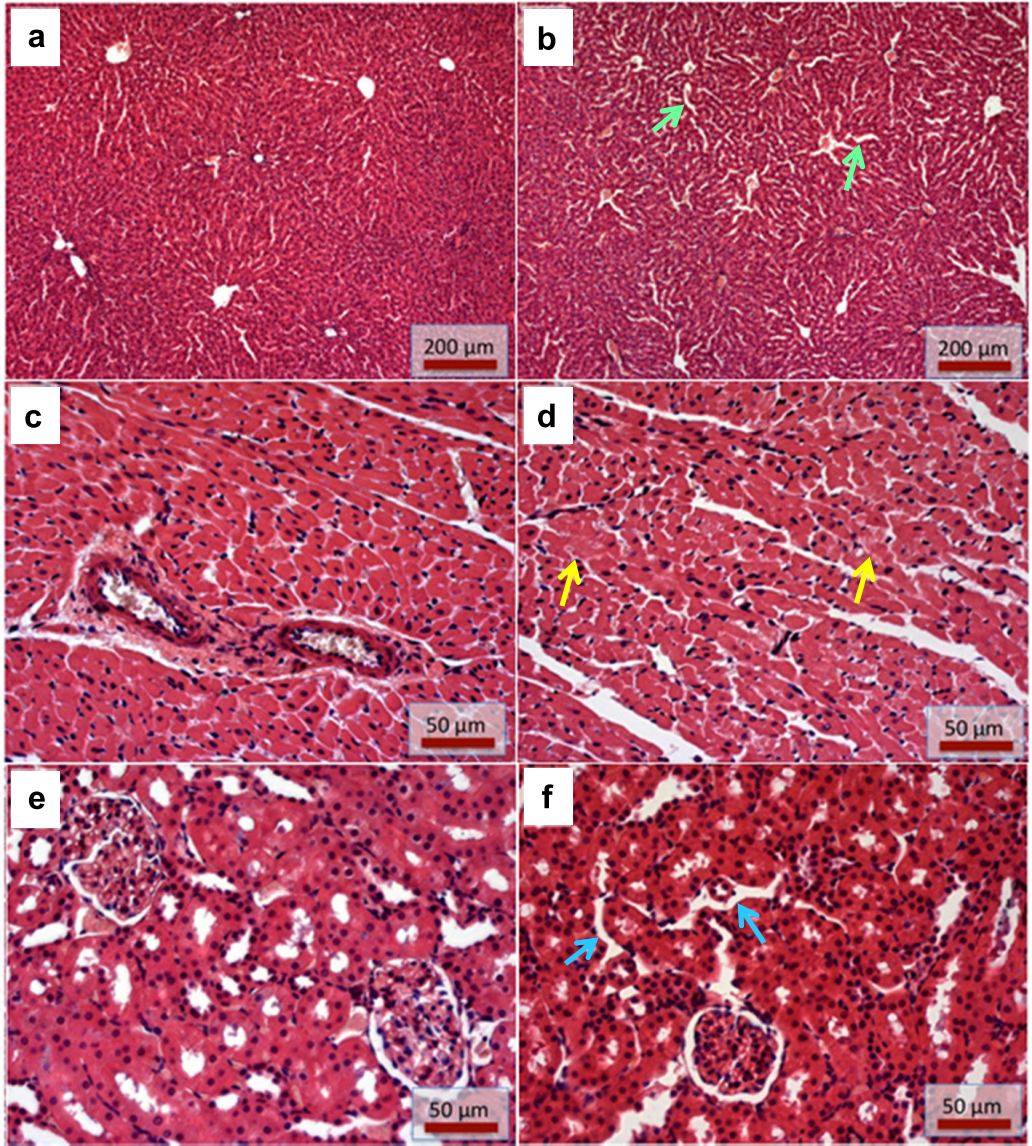
Optical micrographs of tissue sections stained with hematoxylin/eosin. **a** and **b** Photos of liver sections from control (**a**) and MDMA-treated rats (**b**). MDMA-treated rats showed sinusoidal dilatation (*green arrows*) with a marked cellular vacuolization in the periportal regions. **c** and **d** Photos from heart sections of controls (**c**) and MDMA-treated rats (**d**). In **d** scattered cardiomyocytes with signs of intracellular oedema (*yellow arrows*) can be observed, as identified by the reduced cytoplasmic staining. **e** and **f** Photos of kidneys sections from controls (**e**) and MDMA-treated rats (**f**). In **f** a slight interstitial oedema (*blue arrows*), detected by the enlarged space between the tubular structures, can be observed

The qualitative histologic examination of peripheral organs (liver, heart and kidneys) of control and MDMA-treated rats was performed by means of optical microscopy. Representative histological figures can be observed in Fig. 3.

The control group showed a preserved liver tissue structure (Fig. 3a). Livers of MDMA-treated rats presented a marked cellular vacuolization in the periportal regions, and sinusoidal dilatation with periportal and centrilobular vascular congestion (Fig. 3b). No necrotic zones or interstitial inflammatory cell infiltration was observed in either group.

In the histological analysis of the heart, both controls (Fig. 3c) and MDMA-treated (Fig. 3d) animals revealed a normal tissue organization without signs of necrosis or interstitial inflammatory cell infiltration. MDMA exposed animals presented random signs of cardiomyocyte oedema, particularly in the sub-endocardic region (Fig. 3d).

The renal tissue organization remained preserved in the control group (Fig. 3e). However, the MDMA-treated group presented scattered interstitial oedema, detected by the enlarged space between the tubular structures, and signs of vascular congestion (Fig. 3f). No necrotic zones or interstitial inflammatory cell infiltration was observed in either group.

### GSH homeostasis unaffected in the liver, heart, and kidneys

The levels of GSHt, GSSG, GSH and GSH/GSSG ratio in the liver, heart and kidneys in both groups are included in Table 4. No differences were found for all these parameters in the three organs, between control and MDMA-treated animals.

**Table 4:** Effect of MDMA administration in oxidative stress related parameters and ATP levels in the three peripheral organs

Parameter	Control	MDMA
Heart		
GSHt (nmol/mg protein)	9.66 ± 1.30	9.53 ± 1.70
GSH/GSSG ratio	17.62 ± 7.38	15.86 ± 5.93
GSSG (nmol/mg protein)	0.53 ± 0.14	0.59 ± 0.23
GSH (nmol/mg protein)	8.60 ± 1.43	8.35 ± 1.46
Protein carbonylation (% of control)	100.00 ± 13.15	98.94 ± 11.50
ATP (nmol/mg protein)	0.63 ± 0.20	0.60 ± 0.27
Kidneys		
GSHt (nmol/mg protein)	2.25 ± 0.38	1.93 ± 0.60
GSH/GSSG ratio	37.58 ± 11.26	32.39 ± 11.29
GSSG (nmol/mg protein)	0.06 ± 0.02	0.06 ± 0.01
GSH (nmol/mg protein)	2.12 ± 0.34	1.82 ± 0.60
Protein carbonylation (% of control)	100.00 ± 12.55	137.26 ± 37.28
ATP (nmol/mg protein)	1.75 ± 0.62	1.43 ± 0.57
Liver		
GSHt (nmol/mg protein)	19.31 ± 1.96	18.51 ± 2.61
GSH/GSSG ratio	31.46 ± 9.22	28.68 ± 4.46
GSSG (nmol/mg protein)	0.60 ± 0.13	0.62 ± 0.14
GSH (nmol/mg protein)	18.10 ± 2.04	17.28 ± 2.38
Protein carbonylation (% of control)	100.00 ± 27.83	102.61 ± 23.37
ATP (nmol/mg protein)	1.60 ± 0.84	1.69 ± 0.38

Total glutathione (GSHt), oxidized glutathione (GSSG), reduced glutathione (GSH), GSH/GSSG ratio levels, protein carbonylation and ATP levels in heart, kidneys and liver of control and MDMA-treated adolescent rats. Results of GSHt, GSSG, and GSH levels, in nanomol *per* mg of protein (nmol/mg protein), and the GSH/GSSG ratio are presented as mean ± standard deviation, and were obtained from six to seven animals. Statistical comparisons were made using the t-test for GSHt, GSSG, GSH levels in the heart, kidneys and liver, and the GSH/GSSG ratio in the liver; and the Mann-Whitney Rank Sum test for the GSH/GSSG ratio levels in the heart and kidneys. Results of protein carbonylation, in percentage of controls (% of control), are presented as mean ± standard deviation, and were obtained from six to seven animals in each group. Statistical comparisons were made using the t-test for the protein carbonylation in the heart and liver, meanwhile the Mann-Whitney Rank Sum test showed in the kidneys a tendency for a change in protein carbonylation (*p* = 0.051 treatment vs. control). Results of ATP levels, in nmol *per* mg of protein (nmol/mg protein), are presented as mean ± standard deviation, and were obtained from seven animals in each group. Statistical comparisons were made using the t-test for the ATP levels in the heart and kidneys, and the Mann-Whitney Rank Sum test for the ATP levels in the liver

### ATP content was unchanged in the peripheral organs

ATP levels were measured in the liver, heart and kidneys, and no significant differences in the ATP content were observed between control and MDMA-treated animals, as can be seen in Table 4.

### Protein-bound quinones formation increased by MDMA in the liver

**Fig. 4:**
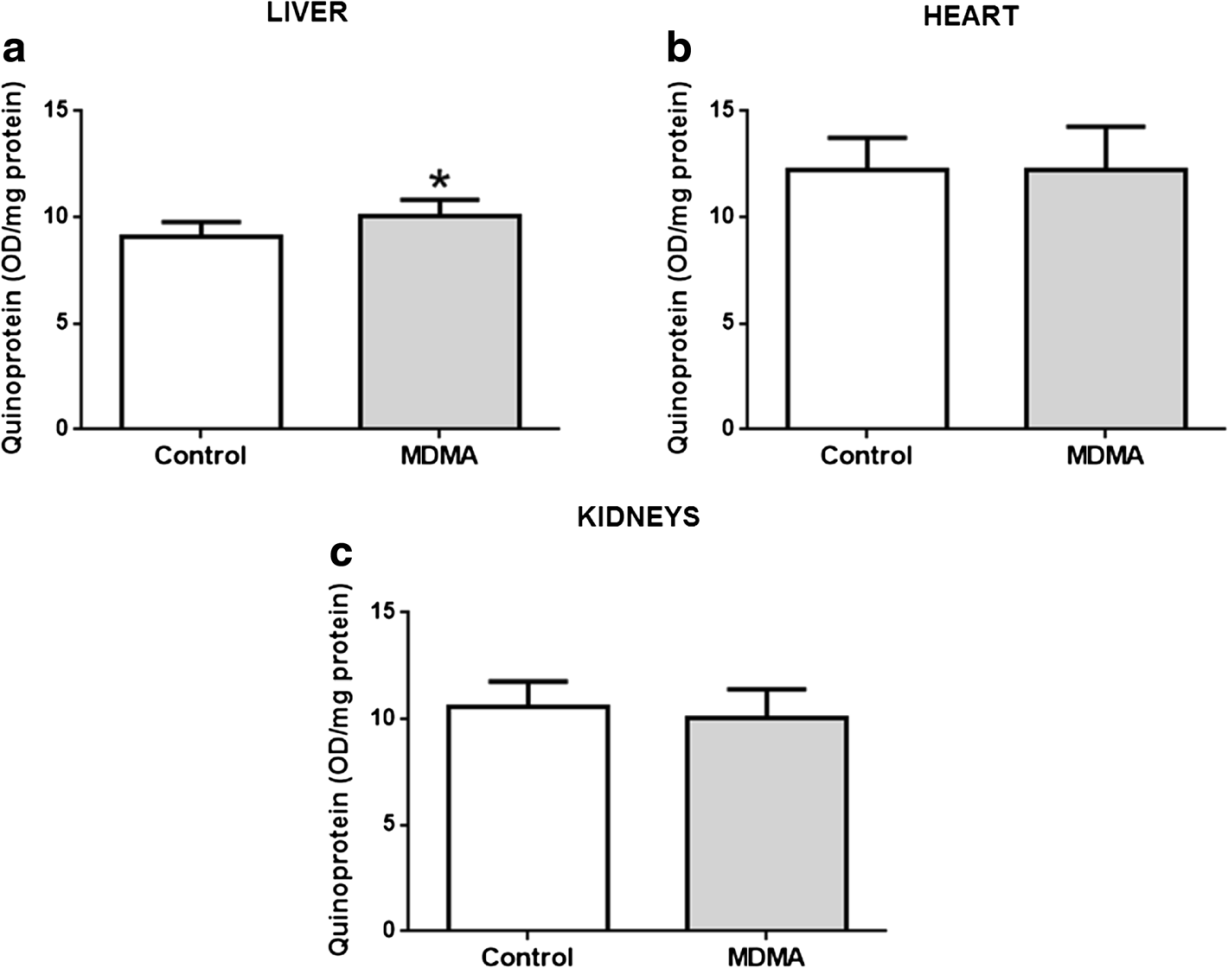
Quinoprotein levels in liver (**a**), heart (**b**) and kidneys (**c**) of control and MDMA-treated adolescent rats. Results, in optical density *per* mg of protein (OD/mg protein), are presented as mean ± standard deviation, and were obtained from seven animals in each group. Statistical comparisons were made using the t-test for the quinoprotein levels in the liver and kidneys and the Mann-Whitney Rank Sum test for the quinoprotein levels in the heart (*p <0.05 treatment vs. control)

In Fig. 4, the levels of protein-bound quinones (quinoproteins) in the liver, heart and kidneys of control and MDMA-treated animals can be observed. There was a significant increase in hepatic quinoprotein levels in the MDMA-treated group (*p* = 0.039) (Fig. 4a). No differences were found regarding this parameter in the heart or kidneys when comparing both groups (Fig. 4b and c).

### MDMA showed a tendency to increase protein carbonylation in the kidneys

In Table 4 are presented the results of protein carbonylation in the liver, heart and kidneys in the two groups. No significant differences were found in the levels of protein carbonyls in the liver and heart between groups. However, data showed a tendency for an increase (*p* = 0.051) in protein carbonylation in the kidneys of MDMA-treated rats, although not reaching statistical significance.

### Caspase-8 activity decreased in the liver, while caspase-3 and -9 activities remained unaltered

Data concerning the activities of caspase-3, -8 and -9 in liver, heart, and kidneys in the two groups are presented in Table 5. Caspase-8 activity significantly decreased in the liver of MDMA-treated rats when compared to controls (*p* = 0.038). No differences were found in the activity of this protease either in heart or kidneys. The activities of caspase-3 and caspase-9 had no significant alterations in all three organs following MDMA when compared to the control group.

**Table 5:** Effect of MDMA administration in caspase-3, -9 and -8 activities in the three peripheral organs

Parameter (FU/μg protein)	Control	MDMA
Heart		
Caspase-3	0.44 ± 0.05	0.42 ± 0.06
Caspase-9	0.09 ± 0.02	0.11 ± 0.05
Caspase-8	0.48 ± 0.08	0.47 ± 0.11
Kidneys		
Caspase-3	0.59 ± 0.08	0.56 ± 0.06
Caspase-9	0.90 ± 0.18	0.79 ± 0.11
Caspase-8	0.66 ± 0.09	0.62 ± 0.07
Liver		
Caspase-3	1.19 ± 0.31	1.12 ± 0.31
Caspase-9	0.90 ± 0.07	0.86 ± 0.13
Caspase-8	1.00 ± 0.06	0.93 ± 0.20*

Caspase-3, -8 and -9 activities in the heart, kidneys and liver of adolescent rats that received either saline or MDMA. Results of caspase-3, -8 and -9 activities, in fluorescent units *per* μg of protein (FU/μg protein), are presented as mean ± standard deviation, and were obtained from seven animals in each group. Statistical comparisons were made using the t-test for the caspase-3 activity in the liver, heart and kidneys and caspase-8 and -9 activities in the heart and kidneys, and the Mann-Whitney Rank Sum test for the caspase-8 and -9 activities in the liver (*p <0.05 treatment vs. control)

## Discussion

In the present work, we studied the acute effects of a moderate MDMA binge dose scheme (3 × 5 mg/kg, every 2 h, i.p.) in an adolescent rat model. Using a dose that does not evoke serotonergic neurotoxicity in adolescent rats we did not observe any signs of brain oxidative stress and the toxic effects occurred mainly in the peripheral organs. The major findings were: 1) sustained hyperthermic response after the second MDMA dose; 2) decreased ATP levels in the frontal cortex; 3) no oxidative stress related changes in the brain areas studied, as no alterations were seen in GSH/GSSG ratio, quinoprotein and protein carbonylation levels; 4) signs of histological damage were seen in the liver, heart, but most notoriously in the kidneys; 5) quinoproteins increased in the liver of MDMA-treated animals.

Animals reproduce the MDMA-induced hyperthermia seen in humans. Neurotoxic doses can promote core body temperatures of 40 °C in rats [27, 35], once again more related to an intensive abuser profile. In PND 40 rats 4 × 10 mg/kg, every 2 h, i.p., can evoke a robust hyperthermia, reaching 2.5 °C above the control levels [27]. In this study, MDMA promoted a significant increase in adolescent rats’ body temperature, rendering a mean increase of about 1 °C. Therefore, we proved that lower MDMA doses do produce hyperthermia in adolescent rats. Our dose scheme clearly more closely reproduces the human scenario, since human studies found body temperature increases of about 1 °C in “ecstasy” dance clubbers [36], thus rendering more reliable the extrapolation to human adolescents.

MDMA abuse promotes several physiological changes, and, for that reason, we also evaluated body weight gain, as well as water and food consumption. In the literature, the cardiovascular changes and anorectic actions of amphetamines and MDMA are well described [2]. A decrease in body weight was found in both groups of animals with similar levels, possibly a consequence of stress evoked by animal handling throughout the experiment. However, water intake slightly increased in MDMA-treated animals in the next 24 h following exposure, most likely due to MDMA-induced hyperthermia. Our acute protocol neither evoked slower weight gain nor animal dehydration, which is seen in protocols of animal MDMA exposure during several days [37]. Therefore, it appears that dehydration has not an important role in our overall results.

Hyperthermia is a well-known important element in MDMA-evoked neurotoxic actions to animals [16], and drugs that prevent MDMA-induced hyperthermia protect against MDMA-induced serotonergic neurotoxicity [35, 38]. Furthermore, studies in vitro in neuronal cultures revealed that the MDMA-induced neurotoxic effects are potentiated by hyperthermia [26, 39]. A report using adult (10 week old) Wistar rats that received the same MDMA binge scheme of our study, revealed that under normal ambient temperature (21.5 °C) animals showed an hyperthermic response and long-term 5-HT depletion, which were blocked when the drug was administrated under low environmental temperature (15 °C) [16]. Therefore there is a tight relation between body temperature and MDMA serotonergic neurotoxicity [16]. Moreover, we have shown that our MDMA regimen (3 × 5 mg/kg, every 2 h, i.p.) in adolescent (PND 40) Wistar rats did not promote serotonergic neurotoxicity 7 days after MDMA exposure (manuscript in preparation). Altogether, our results show that hyperthermia* per se* is not the triggering factor for serotonergic toxicity or to other neurotoxic actions. Nevertheless, the hyperthermia induced by MDMA certainly potentiates the toxicity found in the brain and most importantly in the peripheral organs.

Despite the absence of serotonergic neurotoxicity, we sought to study other markers of brain toxicity, namely energetic status and oxidative stress parameters. It is important to know whether doses that do not cause depletion of monoamines can elicit other toxic brain changes. Several studies found transient changes in ATP brain levels following amphetamines exposure, namely MDMA or methamphetamine (METH). In fact, amphetamines are known to promote changes in tricarboxylic acid cycle’s enzymes function, inhibition of the complexes in mitochondrial electron transport chain and overall interference with mitochondrial dynamics [40]. A binge dose regimen of MDMA (4 × 10 mg/kg, every 2 h, i.p.) with a total dose of 40 mg/kg found decreases in the ATP levels in the striatum at time-points 1, 12 and 24 h, as well as in the hippocampus 12 h after MDMA exposure [41]. A single METH dose of 5 mg/kg, i.p., caused a decline in ATP brain content in adolescent Wistar rats (4 week old) 30 min after administration [42]. The same authors also found that ATP brain levels returned to control values 24 h following exposure to the single METH dose [42]. Most likely there is a possible transitory effect in brain ATP levels caused by MDMA, and the time frame may disclose that short periods following exposure a decrease in ATP levels occurs, meanwhile longer periods following exposure ATP brain levels can recover. Of note that only the frontal cortex area showed a decrease in the ATP levels in the present study. The involvement of this area in both memory and decision-making is widely known, however it is unclear whether this event is related with the impairment in memory and learning abilities seen in young animals exposed to MDMA [7, 8]. Importantly, in humans, “ecstasy” use was related to altered brain activity patterns during associative learning in the left dorsolateral prefrontal cortex [13], an effect that we speculate to be related to MDMA-induced energetic impairment in the frontal cortex. More investigation is needed in adolescent animals to confirm the long-lasting effects of amphetamines in ATP brain levels.

The metabolism of MDMA is a known triggering factor for the toxicity of this drug. The MDMA metabolites are highly reactive and can evoke oxidative stress [1]. Several studies showed that MDMA metabolites promote neurotoxic effects to laboratory animals [43, 44]. Moreover, MDMA metabolites, N-methyl-α-methyldopamine (N-Me-α-MeDA, 3,4-dihydroxymethamphetamine, HHMA) and α-methyldopamine (α-MeDA, 3,4-dihydroxyamphetamine, HHA), induced neuronal death in cultured cells [26, 39, 45]. N-Me-α-MeDA and α-MeDA are important MDMA metabolites that can be oxidized into ortho-quinones, and  that enter a redox cycle-eliciting oxidative stress [1]. Moreover, thioether MDMA metabolites were shown to promote depletion of neuronal GSH and the formation of quinoproteins in cultured neurons [26], and the mixture of MDMA and its metabolites were shown to impair mitochondrial fusion/fission equilibrium and trafficking in cultured neurons [46]. Catechol MDMA metabolites were also shown to promote toxicity to cardiomyocytes [17] and to hepatocytes [47] in vitro. There are other contributing factors for MDMA-induced oxidative stress, including monoamine neurotransmitters metabolism by monoamine oxidase [27], and nitric oxide formation leading to damaging reactive nitrogen species [1].

Our paradigm of MDMA exposure elicited no oxidative stress related changes in the adolescent rat brain. Other studies reported decreases in glutathione levels [48] and increases in protein carbonylation [27] in the rat brain after MDMA exposure. Major differences between the previously mentioned studies and the current study are the use of higher doses, older animals, and different time-points at measurements. The lack of changes in brain oxidative stress parameters following MDMA-induced hyperthermia confirms that hyperthermia induction *per se* is not a guarantee for MDMA-evoked brain oxidative stress.

Regarding the oxidative stress related parameters evaluated in the liver, heart, and kidneys, we could only find an increase in quinoproteins in the liver following MDMA exposure. In fact, MDMA metabolism is primarily hepatic, and, as previously mentioned, promotes the formation of catechol metabolites that can generate protein-bound quinones. Moreover, in rat hepatocytes, catechol MDMA metabolites promoted ortho-quinones formation and oxidative stress [47]. The increase in liver quinoprotein formation possibly reflects the contribution of MDMA metabolism and the formation of reactive metabolites, and reveals the higher susceptibility of the liver to MDMA-evoked toxicity.

Using higher doses and older animals, others reported decreases in glutathione levels. Following administration of a high MDMA dose in a total of 160 mg/kg (20 mg/kg, i.p., 2 daily doses for 4 days), authors found decreases in GSH levels in the rat liver 3 and 6 h following MDMA, which were recovered after 7 days [20, 22]. The heart of rats exposed to MDMA in a dose of 20 mg/kg, i.p., showed a reduction in total GSH levels at the 6 h time-point [22]. The fact that our low MDMA binge dose did not elicit changes in GSH levels in the peripheral organs 24 h following exposure, reveals that GSH levels might not have been affected or that GSH could decrease at early times of exposure but then recovered. Overall, moderate MDMA doses in rats do not seem to elicit long lasting GSH and ATP decreases to the peripheral organs.

Protein carbonylation increase was observed in the rat liver 12 h following two doses of MDMA 10 mg/kg administered orally with a 24 h interval [23]. Enhanced carbonylation in the kidney has been associated with the development of hypertension and kidney disease [49]. Importantly, MDMA administration was shown to increase blood pressure in humans [36], as well as in laboratory animals [4]. In accordance, both the histological damage and the trend for protein carbonylation increase that we found in the kidneys reveal that this organ may be highly prone to damage following MDMA.

Vascular alterations that we observed in the three studied organs, including vascular congestion, after exposure to MDMA have been associated to the MDMA-elicited hyperthermic response [50]. In fact, other studies reported vascular lesions in the peripheral organs as a consequence of hyperthermia [51, 52]. The exposure of Wistar rats to high temperature environments was previously shown to result in several vascular lesions in the heart, liver, kidneys, and lungs of animals that can possibly lead to functional organ failure [52]. Those effects were similar to the signs of damage observed in our study. Halpin and co-workers reported morphological damages in the liver of rats 24 h after the treatment with METH. The referred morphological changes were prevented when the hyperthermic response induced by the treatment with METH was blocked, suggesting that liver damage is possibly a consequence of METH-induced hyperthermia [51]. Therefore, it can be postulated that the hyperthermic response observed in our experiment may have contributed to the observed histological alterations. The liver may be an organ with greater susceptibility to MDMA toxicity as indicated not only by the tissue damage, but also because it was the only to show an increase in quinoprotein formation. Of note, that one of the most frequently reported damage promoted by MDMA in humans is hepatotoxicity [18, 24, 25]. In fact, MDMA liver metabolism and hyperthermia may cooperate to render the liver very vulnerable to damage.

The lack of changes in the plasma levels of AST, ALT, CK and CK-MB after MDMA exposure corroborates the absence of necrosis in the heart and liver. Moreover, the absence of caspase activity increase in the organs proves the lack of severe damage to the tissues, given that caspases are important effectors of the apoptotic pathway [53]. The notable exception of caspase-8 activity in the liver, which revealed a significant decrease in MDMA-treated rats, might be related to a repression of genes related to apoptosis. The inhibition of caspase-8 activity has been observed in hepatic cells through nitric oxide signalling [54]. In cultured rat striated cardiac myocytes there was a repression of caspase-1 and caspase-8 genes following exposure to MDMA [55]. Using higher doses (20 mg/kg, i.p., twice daily during 4 days, in a total dose of 160 mg/kg), hepatic necrosis with inflammatory infiltrate around hepatic vein and increases in AST levels were found at the 6 h time-point [20]. The heart is particularly susceptible to oxidative stress-related injuries and amphetamines like MDMA evoke cardiotoxicity [56]. Exposure of adult Sprague-Dawley rats (300 g) to MDMA binges (9 mg/kg, intravenous twice daily, for 4 days), a neurotoxic dose regimen, showed that MDMA promoted cardiac foci of inflammatory infiltrates, the presence of necrotic cells and/or disrupted cytoarchitecture [4]. Another report in adult rats (200–250 g) after a single MDMA dose (20 mg/kg, i.p.) showed myocardial necrosis and following 16 h the heart exhibited macrophagic monocytes around the necrotic myocardial cells [22]. Our study used lower MDMA doses in adolescent rats and drug exposed hearts showed a particular vulnerability of the myocytes from the sub-endocardic region. Reports from MDMA users following fatalities describe major organ changes, including necrosis, oedema and inflammation [1, 24, 25]. These important human findings report a rather extreme scenario following the course of MDMA intoxication. Our report more reliably reproduces the hyperthermia seen in human abusers and organ changes might be more similar to those seen in adolescents.

## Conclusions

MDMA moderate doses, more close to those used by human adolescents, do not elicit in adolescent rats oxidative stress related changes in the brain. Meanwhile, ATP levels in frontal cortex were decreased following MDMA. MDMA treatment in adolescent rats promoted morphological tissue alterations in the heart, kidneys, and liver, as well as rises in liver quinoproteins. New studies are required to assess the impact of moderate MDMA doses in adolescent animals to verify whether these brain and peripheral organ changes are long-lasting and may be reflected later during adulthood.

## Abbreviations

5-HT, 5-hydroxytryptamine, serotonin; ALT, alanine aminotransferase; AST, aspartate aminotransferase; ATP, adenosine 5′-triphosphate; CK, total creatine kinase; CK-MB, creatine kinase-MB; EDTA, ethylenediaminetetraacetic acid; GSH, reduced glutathione; GSHt, total glutathione; GSSG, oxidized glutathione; h, hour; HEPES, 4-(2-hydroxyethyl)piperazine-1-ethanesulfonic acid; i.p., intraperitoneal; KHCO_3_, potassium bicarbonate; MDMA, 3,4-methylenedioxymethamphetamine or “ecstasy”; METH, methamphetamine; min, minutes; NaCl, sodium chloride; N-Me-α-MeDA, N-methyl-α-methyldopamine, 3,4-dihydroxymethamphetamine, HHMA; PBS, phosphate buffered saline; PMSF, phenylmethanesulfonyl fluoride; PND, postnatal day; α-MeDA, α-methyldopamine, 3,4-dihydroxyamphetamine, HHA
